# Untargeted HILIC-MS-Based Metabolomics Approach to Evaluate Coffee Roasting Process: Contributing to an Integrated Metabolomics Multiplatform

**DOI:** 10.3390/molecules25040887

**Published:** 2020-02-17

**Authors:** Raquel Pérez-Míguez, María Castro-Puyana, Elena Sánchez-López, Merichel Plaza, María Luisa Marina

**Affiliations:** 1 Departamento de Química Analítica, Química Física e Ingeniería Química, Universidad de Alcalá, Ctra. Madrid-Barcelona Km. 33.600, 28871 Alcalá de Henares (Madrid), Spain; r.perezm@edu.uah.es (R.P.-M.); maria.castrop@uah.es (M.C.-P.); elena.sanchezl@edu.uah.es (E.S.-L.); merichel.plaza@uah.es (M.P.); 2 Instituto de Investigación Química Andrés M. del Río (IQAR), Universidad de Alcalá, Ctra. Madrid-Barcelona Km. 33.600, 28871 Alcalá de Henares (Madrid), Spain

**Keywords:** coffee roasting process, HILIC, mass spectrometry, multiplatform, untargeted metabolomics

## Abstract

An untargeted metabolomics strategy using hydrophilic interaction chromatography-mass spectrometry (HILIC-MS) was developed in this work enabling the study of the coffee roasting process. Green coffee beans and coffee beans submitted to three different roasting degrees (light, medium, and strong) were analyzed. Chromatographic separation was carried out using water containing 10 mM ammonium formate with 0.2 % formic acid (mobile phase A) and acetonitrile containing 10 mM ammonium formate with 0.2 % formic acid (mobile phase B). A total of 93 molecular features were considered from which 31 were chosen as the most statistically significant using variable in the projection values. 13 metabolites were tentatively identified as potential biomarkers of the coffee roasting process using this metabolomic platform. Results obtained in this work were complementary to those achieved using orthogonal techniques such as reversed-phase liquid chromatography-mass spectrometry (RPLC-MS) and capillary electrophoresis-mass spectrometry (CE-MS) since only one metabolite was found to be common between HILIC-MS and RPLC-MS platforms (caffeoylshikimic acid isomer) and other between HILIC-MS and CE-MS platforms (choline). On the basis of these results, an untargeted metabolomics multiplatform is proposed in this work based on the integration of the three orthogonal techniques as a powerful tool to expand the coverage of the roasted coffee metabolome.

## 1. Introduction

Coffee is a valuable and highly consumed drink over the world due to its interesting organoleptic characteristics [1]. The main compounds found in coffee comprise alkaloids, phenolic compounds, carbohydrates, amino acids, proteins, and lipids, being some of them bioactive and having beneficial health effects, such as chemopreventive, antihypertensive, hypoglycemic, antiglycative, and anticarcinogenic [2]. Generally, the most abundant coffee bioactive compounds are caffeine, chlorogenic acids, and trigonelline followed by diterpene alcohols, such as cafestol and kahweol, and non-digestible fiber. Additionally, the coffee roasting process contributes to the formation of another class of bioactive compounds called melanoidins, which are Maillard reaction products [3]. Hence, there are many works in the literature focused on the study of these bioactive coffee compounds [2]. However, not many studies have been carried out using a metabolomics approach although this omic strategy is considered a powerful tool in food analysis. Metabolomics is focused on the analysis of the metabolome of a certain biological system which is composed of molecules with a molecular mass below 1500 Da. For example, this strategy has been employed for the discrimination of coffee varieties and geographical origins [4,5,6,7,8,9,10,11,12,13], caffeinated and decaffeinated coffees [14], and ground roasted and instant coffees [15].

Metabolomics has also been applied to investigate the changes on the chemical composition taking place during the coffee processing as a result of pyrolysis and Maillard reactions, among others. These changes can have influence on the coffee quality and safety, which makes crucial the study of markers capable of discriminating the changes occurring during the roasting. For instance, different methods employed in coffee brewed have been evaluated because they can affect the metabolomic profile [5,16,17]. In this line, nuclear magnetic resonance (NMR) spectroscopy-based metabolomics and human sensory test were used to study the chemical compounds enabling to distinguish and predict the different sensations of coffee taste [18,19]. On-line analysis of coffee samples submitted to roasting was carried out by ion mobility spectrometry-mass spectrometry (IMS-MS) to monitor the formation of volatile compounds and the release of fatty acids [20]. Additionally, targeted and non-targeted analysis by mass spectrometry (MS), two-dimensional gas chromatography (GC × GC)-MS and GC-MS were carried out for understanding the role of chlorogenic acids and the volatile fraction during coffee roasting process, respectively [21,22,23] and a non-target and non-volatile approach based on the use of ambient technique (EASI) coupled to a single quadrupole MS was employed to monitor roasting chemical changes in the coffee bean [24].

Our research team has investigated for the first time the coffee roasting process using untargeted metabolomics strategies based on RPLC-MS and CE-MS [25,26] and different metabolites were proposed as biomarkers of this process. The hypothesis of the current work relies on what markers of coffee roasting could be obtained using a HILIC-MS strategy. To this end, an untargeted metabolomics approach based on the use of HILIC-MS as analytical platform is proposed to achieve the metabolomic analysis of green coffee and coffee samples submitted to different roasting degrees. The results obtained were compared with those previously achieved using RPLC-MS and CE-MS untargeted metabolomics platforms. This study revealed the potential of a metabolomic multiplatform consisting of different liquid-phase orthogonal techniques to expand the coverage of the metabolome of a given system such as roasted coffee.

## 2. Results and Discussion

### 2.1. Metabolomics Analysis of Coffee Samples by HILIC-MS

In order to achieve the metabolomics analysis of coffee samples submitted to different roasting degrees, a method based on the coupling HILIC-MS was tuned for the given samples. Mobile phases consisting of water containing 0.1 % formic acid (solvent A) and acetonitrile containing 0.1 % formic acid (solvent B) were assayed using an elution gradient 98–55% B in 45 min; 55% B during 4 min; 55–98% in 2 min. However, under these conditions, positive ionization did not enable the MS detection of the metabolites although 162 molecular features were detected in negative ionization mode. Therefore, water containing 0.2 % formic acid with 10 mM ammonium formate (solvent A) and acetonitrile containing 0.2 % formic acid with 2 mM ammonium formate (solvent B) were employed using the same elution gradient. These mobile phases also showed to give good metabolite coverage for food and biological samples in previous works by our research team [27,28]. Under these conditions, the ionization was successful, and 280 and 223 molecular features were detected in positive and negative modes, respectively. All these experiments were carried out using an injection volume of 5 µL (since using 10 µL the number of molecular features decreased) and a flow rate of 0.2 mL/min at a temperature of 30 °C. Under these conditions, the effect of the column temperature on the number of molecular features was investigated (30, 40, and 50 °C). The results obtained showed that a decrease in the number of molecular features was observed when increasing the temperature both in positive and in negative ionization modes so that a temperature of 30 °C was chosen as optimum. Finally, a flow rate of 0.3 mL/min was assayed for comparison but a decrease in the number of molecular features was obtained. So, a flow rate value of 0.2 mL/min was employed for further experiments. Although initially a sheath gas temperature of 300 °C, a sheath flow of 6.5 L/min and a fragmentator voltage of 175 V were employed as MS conditions, other values for these variables were also assayed (250 °C, 5.5 L/min and 125 V, respectively) without improving the number of features obtained. Figure S1 shows the base peak chromatograms obtained for green coffee and for coffee samples submitted to three roasting degrees under the above-mentioned chromatographic and MS conditions in the positive ionization mode.

**Figure 1:**
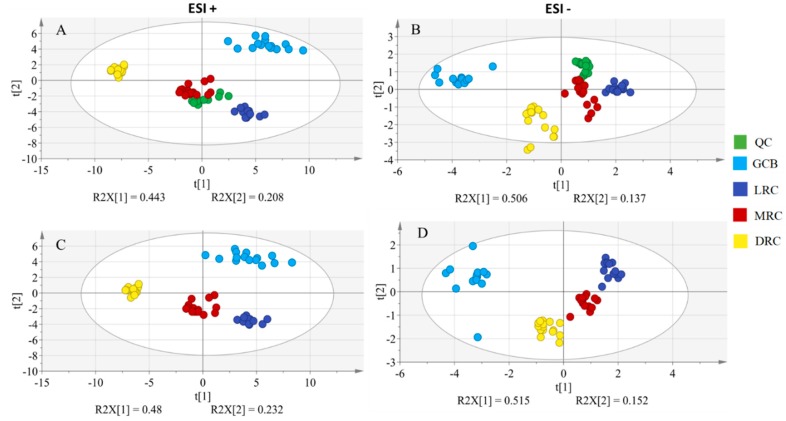
Principal component analysis (PCA) score plots obtained in positive and negative ionization modes for the four studied coffee groups (GCB, LRC, MRC, and DRC) submitted to different roasting degree with QC samples (**A**,**B**) and without QC samples (**C**,**D**).

Using the optimal conditions for HILIC-MS method, metabolomic sequence was acquired as detailed in section “3.4 metabolomics sequence”. 53 and 40 molecular features were the resulting number of features for positive and negative ionization modes sequences, respectively. PCA score plots having using molecular features as variables and the samples as observations displayed good QC and sample grouping clustering both for positive (Figure 1A) and negative (Figure 1B) ionization modes. Score plots of PCAs models excluding the QC samples revealed similar pattern in sample clustering meaning that plots are not influenced by QC samples in both positive (Figure 1C) and negative (Figure 1D) ionization modes. Variance explained in first two components was similar for both ionization modes, 48 and 23 % for ESI+ (Figure 1C) and 51% and 15 % for ESI- (Figure 1D). Note that in the ESI+ separation between the unroasted samples (GCB) and the roasted samples (LRC, MRC, and DRC) was in the second component, whereas that in the ESI- was in the first component. Focusing only on the roasting degree, there was a gradual trend, which could be observed both in first and second components for both ionization modes.

To further explore these data, another type of unsupervised multivariate analysis was conducted, i.e., hierarchical clustering analysis (HCA) (see Figure S2). As anticipated in the PCA score plots, there was a good clustering also observed in HCA. In the positive ionization mode (Figure S2A) the most difference was found between DRC and the rest of samples while that in the negative mode (Figure S2B) the GCB was most different group of samples. This is in line with the results from Figure 1 since DRC (in ESI+) and GCB (in ESI-) samples are further away from the rest of samples on the first component. Both PCA and HCA plots support the existence of differences in the metabolic profiles of the coffee samples upon different roasting degrees.

**Figure 2:**
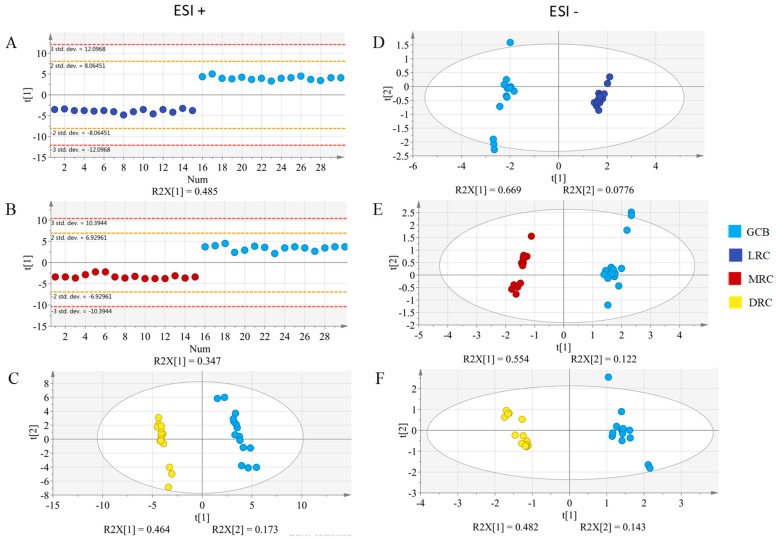
Partial least squares discrimination analysis (PLS-DA) score plots for LRC, MRC, and DRC compared with GCB in positive (**A**, **B**, and **C**, respectively) and in negative (**D**, **E**, and **F**, respectively) ionization modes.

As the aim of this work is to find markers of roasting degree for coffee, supervised PLS-DA models were used in a pair-wise manner to highlight what metabolic features were significant (Figure 2). Specifically, these pair-wise comparisons included matching GCB vs. each roasting degrees (LRC, MRC and DRC). Quality parameters (R^2^X, R^2^Y, and Q^2^) and results of the cross-validated ANOVA (F, and *p*-values) for both ionization modes are listed on Table 1. Given the good cluster found in the unsupervised models (PCA), it is not surprising to see the good R^2^ and Q^2^ values as well as the very high values for the cross-validation ANOVA in the supervised PLS-DA models. Both ionization modes behaved in a very similar manner. This highlights the fact that for the given HILIC-based methodology both ionization modes can be used to extract metabolic profiling in a reliable manner. Moreover, as will be further discussed in the next section, using both ionization modes is very advantageous as it increases the metabolite coverage.

### 2.2. Metabolite Identification of Coffee Roasting Process

In order to study the metabolites involved in the coffee roasting process, an exhaustive identification focused on the metabolic features with VIP values for the pair-wise PLS-DA models higher than 1.0 in the positive and negative ionization modes was carried out. Based on this criterium, 20 and 13 molecular features were selected in positive and negative ionization modes, respectively. Table 2 (negative mode) and Table 3 (positive mode) summarize the retention time, the molecular feature, the experimental *m*/*z* value, the mass error comparing with the database, the main fragments obtained in MS/MS spectra, the VIP values for the pairwise PLS-DA models, and the trend observed for all significant metabolites along the roasting process of the coffee (Figure S3 shows the diagrams of the trends observed for all tentatively and unequivocally compounds along the coffee roasting process). As it can be seen in both tables, seven and six metabolites were identified in negative and positive ionization modes, respectively. From the 13 identified metabolites, 5 were unequivocally identified.

In the negative ionization mode, mainly different kind of phenolic compounds were identified such as hydroxycinnamic acids (such as neochlorogenic acid and hydroxycinnamic acid methyl ester derivative), hordatines (such as hordatine A1 and hordatine A1 hexose isomers), caffeoylshikimic acid isomer, and ssioriside (see Table 2). In general, the trend of the identified phenolic compounds is to decrease as roasting process increases (except in the case of hydroxycinnamic acid methyl ester derivative) because they are thermolabile and easily decompose under the effect of high temperature resulting in their degradation [29].

On the other hand, in the positive ionization modes, choline and the amino acids betaine, proline, and the proline betaine were unequivocally identified, while *N*-methylpipecolic acid and 2-methyl-(methylthiol)pyrazine isomer were tentatively identified (see Table 3). These markers decreased during roasting process. All the identified compounds grouped in Table 2 and Table 3 had been previously described in coffee samples in the literature [25,26,30,31,32,33].

### 2.3. Integration of the Untargeted HILIC-MS Strategy Developed in a Metabolomics Multiplatform for the Search of Markers of the Coffee Roasting Process

**Figure 3:**
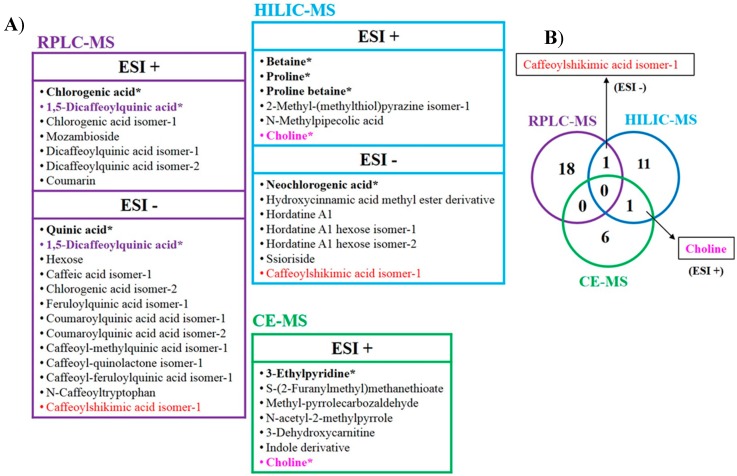
(**A**) List of metabolites tentatively identified by the integrated untargeted metabolomics multiplatform based on HILIC-MS, RPLC-MS [25] and CE-MS [26]. Those metabolites common in two platforms are highlighted using different colors. Metabolites unequivocally identified for each platform by the injection of standards (matched retention/migration times and MS/MS spectra fragmentation) are marked in bold and with an asterisk. (**B**) Venn diagram displaying the total number of different metabolites tentatively identified by the untargeted multiplatform proposed and those common to two platforms.

Results obtained by the HILIC-MS metabolomics strategy developed in this work complement those obtained by our research team using RPLC-MS [25] and CE-MS [26], contributing to enlarge the coverage of the roasted coffee metabolome in the study of the coffee roasting process. In fact, the use of three orthogonal analytical techniques such as RPLC-, HILIC-, and CE-MS, constitutes a powerful untargeted metabolomics multiplatform integrated by these three techniques that originate complementary information. Figure 3A shows a comparative among the metabolites tentatively identified by each of the developed strategies (19 by RPLC-MS, 13 by HILIC-MS, and 7 by CE-MS). Moreover, this figure indicates which of these metabolites are unequivocally identified (marked in bold and with an asterisk) by the injection of standards (matched retention/migration times and MS/MS spectra fragmentation) showing that a total of nine different metabolites were unequivocally identified using the proposed multiplatform. On the other hand, the Venn diagram shown in Figure 3B illustrates the potential of using the integrated platform developed since just one metabolite was common between RPLC-MS and HILIC-MS platforms (isomer of caffeoylshikimic acid) and another between HILIC-MS and CE-MS platforms (choline) while there were not common metabolites found by RPLC-MS and CE-MS platforms. These results demonstrate the relevance of using orthogonal techniques to provide information on the whole metabolome including compounds of diverse characteristics. They also show that some isomers of the same compound (e.g., dicaffeoylquinic acids or coumaroylquinic acid) with the same *m*/*z* ratio but different retention times were found although they could not be distinguished in this work.

Moreover, as both ionization modes were employed for RPLC-MS and HILIC-MS analysis (analysis by CE-MS could only be achieved in ESI+), they were shown to provide interesting complementary information. Thus, some metabolites with the same nominal mass were found by RPLC-MS in both ionization modes (one of them was unequivocally identified as 1,5-dicaffeoylquinic acid) and were identified as hydroxycinnamic acids. After analyzing the MS/MS fragmentation of these compounds, just one of them was common in RPLC-MS and HILIC-MS platforms (isomer of caffeoylshikimic acid). This compound exhibited the same trend along the roasting process when analyzed by both platforms. In addition, the content of this compound seemed to be relatively stable whereas de contents of other compounds from the same family decreased, all these results confirming the consistency of the results obtained regardless the analytical platform employed. In fact, the levels of many compounds decreased with the roasting process (chlorogenic acids) whereas other formed as products of Maillard reaction increased (3-ethylpyridine, methyl-pyrrolecarboxaldehyde or *N*-acetyl-2-methylpyrrole).

## 3. Materials and Methods

### 3.1. Chemicals and samples

Methanol, acetonitrile, and formic acid of MS-grade were supplied from Fisher Scientific (Hampton, New Hampshire, USA). Ammonium formate of MS grade, 4-*O*-caffeoylquinic acid, quinic acid, shikimic acid, 3-hydroxycoumarin, 7-hydroxycoumarin, trans-caffeic, caffeic acid, trans-ferulic acid, chlorogenic acid, neochlorogenic acid 1-aminocyclohexanecarboxylic acid, methyl benzoate, betaine, norvaline, valine, proline, and choline were purchased from Sigma (St. Louis, MO, USA). Ultrapure water was generated with a Milli-Q system (Millipore, Madrid, Spain).

Green coffee beans (GCB) of the Arabica variety were roasted to light (LRC), medium (MRC), and dark (DRC) levels at 175, 185, and 195 °C during 12.36, 14.11, and 17.06 min, respectively. In order to control the roasting process, the weight loss of each sample was checked, being 13% in light coffee, 15% in medium coffee, and 17% in dark coffee. All these coffee samples were kindly donated, roasted, and grounded by “Café Fortaleza” (Vitoria, Spain) and were identical to the samples analyzed in our previous works using RPLC-MS [25] and CE-MS [26] platforms.

### 3.2. Sample Preparation

Metabolite extraction from grounded coffee samples was carried out following the procedure previously optimized by our research group [25]. Briefly, 1.5 mL of methanol/water (25/75, *v*/*v*) were added to 50 mg of grounded coffee. The extraction was performed using a Thermomixer Compact (Eppendorf AG, Hamburg, Germany) at 1× g during 15 min at room temperature (25 °C). After centrifugation (1137× g, 25 °C, 10 min) the supernatant was collected and injected in the system.

In order to perform the metabolomics sequence, the extractions of each group of coffee samples (GCB, LRB, MRB, and DRB) were carried out five times independently (*n* = 5).

On the other hand, equal amounts of each grounded coffee sample were combined to prepare the quality control (QC) sample.

### 3.3. HILIC-MS Analysis

Metabolic profiling of coffee samples was carried out using a High Performance Liquid Chromatography (HPLC) system 1100 series from Agilent (Agilent Technologies, Palo Alto, CA, USA) coupled to a high sensitive quadrupole time-of-flight mass spectrometer (QTOF/MS) 6530 series (Agilent Technologies, Germany) equipped with a Jet Stream thermal orthogonal electrospray ionization (ESI) source. Agilent Mass Hunter Qualitative Analysis software (B.07.00) was employed for MS control, data acquisition, and data analysis.

The chromatographic method included the use of a HILIC (OH5) Ascentis Express column (100 × 2.1 mm, 2.7 µm particle size with fused core^®^ particles with 0.5 µm thick porous shell) protected by a HILIC (OH5) Ascentis Express guard column (0.5 cm × 2.1 mm, 2.7 μm particle size), both from Supelco (Bellefonte, PA, USA). The mobile phases consisted of water with 0.2 % formic acid and 10 mM ammonium formate (solvent A), and acetonitrile with 0.2 % formic acid and 2 mM ammonium formate (solvent B) in a gradient elution analysis programed as follows: 98–55% B in 45 min; 55% B during 4 min; 55–98% in 2 min; and then the column was re-equilibrated for 15 min using the initial solvent composition. The mobile phase flow rate, column temperature and injection volume were 0.2 mL/min, 30 °C, and 5 µL, respectively.

MS analyses were performed both in positive and negative ESI modes with the mass range set at *m*/*z* 100–1700 (extended dynamic range) in full scan resolution mode at a scan rate of 2 scans per second. ESI parameters for the mass spectrometer were: gas temperature, 300 °C; drying gas flow, 10 L/min; capillary voltage, 3000 V with a nozzle voltage of 0 V; nebulizer pressure, 25 psi; and sheath gas flow and temperature of jet stream, 6.5 L/min and 300 °C, respectively. The fragmentator voltage was set at 175 V whereas the skimmer and octapole voltages were 60 and 750 V, respectively. For MS/MS experiments, the selected precursor ions were fragmented by applying voltages between 20 and 40 V in the collision chamber.

In order to obtain proper mass accuracy, spectra were corrected using ions *m*/*z* 121.0508 (C_5_H_4_N_4_) and 922.0097 (C_18_H_18_O_6_N_3_P_3_F_24_) in ESI positive, and *m*/*z* 112.9856 (C_2_F_3_O_2_) and 966.0007 (C_18_H_18_O_6_N_3_P_3_F_24_) in ESI negative. To achieve this task, a solution from Agilent Technologies containing those ions was continuously pumped into the ionization source at a 15 µL/min flow rate using a 25 mL Gastight 1000 Series Hamilton syringe (Hamilton Robotics, Bonaduz, Switzerland) on a NE-3000 pump (New Era Pump Systems Inc., Farmingdale, NY, USA).

### 3.4. Metabolomics Sequence

In order to guarantee great stability and repeatability of the chromatographic system, blanks and QC samples were injected at the beginning of the metabolomics sequence. Then, a total of 60 coffee samples (five replicates of each group injected in triplicate) were randomly injected and a QC sample was injected every six coffee samples.

### 3.5. Data Processing and Multivariate Analysis

In order to get the data related to the molecular features present in each sample, Molecular Feature Extraction (MFE) tool from Mass Hunter Qualitative Analysis (B.07.00) was employed. The “small molecules (chromatographic)” algorithm was utilized to select MFE extraction following parameters: ions ≥ 500 counts; peak spacing tolerance = 0.0025 *m*/*z*, plus 7.0 ppm; isotope model = common organic molecular; and limited assigned change was set to 2. H^+^, Na^+^, K^+^, and NH^4+^ adducts were considered in positive ionization, whereas that only the HCOO^−^ adduct was chosen for negative ionization to identify different ion species coming from the same molecular feature.

Agilent Mass Profiler Professional (MPP) software (B.02.00) was used to carry out the filtering and alignment of the extracted molecular features. Molecular feature filtering was achieved employing a minimum absolute abundance of 10.000 counts; number of ions 2 and all charges permitted. On the other hand, molecular feature alignment was performed using a retention time window of 0.5 and 3.0 min for positive and negative mode respectively, a mass tolerance of 0.02 Da and a mass window of 15 ppm. Molecular features present in 80 % of all injected QC samples with a coefficient of variation below 30 % were retained for further data analysis to clean data matrix from background signals.

The data were centered and divided by the square root of the standard deviation as scaling factor (Pareto scaling) using multivariate statistical analysis with SIMCA 14.0 software (MSK Data Analytics Solutions, Umeå, Sweden). Firstly, unsupervised principal component analysis (PCA) was employed to check clustering existing in the analyzed samples. Thereupon, partial least squares discriminant analysis (PLS-DA) was applied to discriminate samples according to their roasted degree. The quality of the models was evaluated by the parameters R^2^X, R^2^Y and Q^2^. R^2^ is the explained variability of the model, i.e., goodness of fit, whereas that Q^2^ describes the goodness of prediction, this is, the predictive ability of the model.

### 3.6. Metabolite Identification

The potential markers of roasting degree of coffee were found by comparing models in a pair-wise manner: GCB vs. LRC, GCB vs. MRB, and GCB vs. DRB. Only features with variable importance in the projection (VIP) values of the first component of the PLS-DA models higher or equal than 1.0 were considered as significant and were subjected to the identification process. Metabolite identification was carried out by comparing the obtained accurate mass values in the CEU Mass Mediator (in which the search of metabolites is performed in different databases such as KEGG, METLIN, LipidMass, and HMDB) [34] and in the FoodDB database (http://foodb.ca/) (an error of 30 ppm was considered).

If the standard compounds could be commercially acquired, they were analyzed under identical analytical conditions to obtain their retention times and MS/MS fragmentation patterns in order to confirm the metabolite identity. However, if the standards could not be acquired because they were not commercially available, experimental MS/MS spectra obtained for each molecular feature were compared to those described in HMDB database, literature, and/or predicted MS/MS spectra obtained in CFM-ID (cfmid.wishartlab.com).

## 4. Conclusions

An untargeted metabolomics strategy using HILIC-MS was developed in this work aimed to study the coffee roasting process. Both positive and negative ionization modes were employed which originated 53 and 40 molecular features, respectively, to be statistically analyzed. Using VIP values, 20 and 13 variables were chosen as the most statistically significant metabolites in ESI+ and ESI-, respectively, from which 13 were tentatively identified using this platform (6 in the positive ionization mode and 7 in the negative ionization mode). Five of these metabolites were unequivocally identified through the injection of standards (betaine, proline, proline betaine, choline, and neocholorogenic acid). Results obtained in this work enabled to us reveal the trend followed by statistically significant metabolites with the coffee roasting process as samples submitted to light, medium, and strong roasting were analyzed and compared to green coffee. The levels of some compounds decreased with the roasting process, as expected, while other originated by Maillard reaction increased.

The comparison of the results obtained in this work by the HILIC-MS strategy developed with those achieved when using RPLC-MS and CE-MS strategies showed that just one metabolite was common to HILIC-MS and RPLC-MS platforms (caffeoylshikimic acid isomer) and another to HILIC-MS and CE-MS platforms (choline) demonstrating the big potential of an integrated untargeted metabolomics multiplatform based on the three orthogonal techniques employed in this work to enlarge the coverage of the roasted coffee metabolome and to obtain complementary results, making possible a deeper characterization of the coffee roasting process.

## Figures and Tables

**Table 1:** PLS-DA models for samples submitted to different roasting degrees compared with green coffee.

	ESI+		ESI−	
	Quality Parameters	F (and *p*-values) of Cross-Validated ANOVA	Quality Parameters	F (and *p*-values) of Cross-Validated ANOVA
GCB vs LRC	R^2^X = 0.485 R^2^Y = 0.989 Q^2^ = 0.977	561.6 (1.0 × 10^−22^)	R^2^X = 0.747 R^2^Y = 0.995 Q^2^ = 0.987	466.0 (2.3 × 10^−22^)
GCB vs MRC	R^2^X = 0.347 R^2^Y = 0.972 Q^2^ = 0.903	125.7 (2.1 × 10^−14^)	R^2^X = 0.676 R^2^Y = 0.993 Q^2^ = 0.980	220.2 (1.5 × 10^−18^)
GCB vs DRC	R^2^X = 0.637 R^2^Y = 0.990 Q^2^ = 0.980	197.1 (1.6 × 10^−18^)	R^2^X = 0.625 R^2^Y = 0.988 Q^2^ = 0.962	120.8 (1.6 × 10^−15^)

**Table 2:** MS/MS fragmentation of potential markers of coffee roasting process in negative ionization mode.

							VIP Values			
#	RT (min)	Molecular Formula	Tentative Identification	[M−H]^−^	Mass Error (ppm)	Main MS/MS Fragments	GCB vs. LRC	GCB vs. MRC	GCB vs. DRC	Roasting Trend
**1**	2.2	C_10_H_10_O_4_	Hydroxycinnamic acid methyl ester derivative	193.0483	11	65.0388 121.0280 93.0334 133.0277	0.78670	1.12365	1.49312	↑
**2**	2.5	C_16_H_16_O_8_	Caffeoylshikimic acid isomer	335.0708	11	161.0238 135.0440 179.0333	1.81873	2.19334	2.41682	↓
**3**	2.7	C_28_H_38_N_8_O_5_	Hordatine A1	565.2902	11	59.0136 101.0589 113.0234	1.82774	2.19590	1.86040	↓
**4**	13.2	C_34_H_48_N_8_O_10_	Hordatine A1 hexose isomer	727.3413	16	643.2944 113.0236	0.81788	0.95373	0.94533	↓
**5**	14.9		Unknown	481.2371		59.0133 89.0235	2.41084	2.85921	3.12239	↓
**6**	14.9	C_34_H_48_N_8_O_10_	Hordatine A1 hexose isomer	727.3413	16	643.2942 113.0235 89.0235	2.32700	2.67836	2.78861	↓
**7**	16.4	C_27_H_38_O_12_	Ssioriside	553.2225	3	44.9983 89.0239 59.0143 391.0400 119.0358	1.54553	2.49650	2.52013	-
**8**	17.7		Unknown	135.0436		67.0185	1.38856	1.31171	0.51246	↓
**9**	18.5		Unknown	135.0448		134.0358 89.0381	1.75153	1.26031	1.05637	↓
**10**	18.6	C_16_H_18_O_9_	Neochlorogenic acid *	353.0803	7	191.0553 179.0339 135.0439	2.04079	1.59240	0.56977	↓
**11**	21.6		Unknown	705.3292		659.3259 335.2215 323.0965	1.80217	9.9 × 10^−9^	9.7 × 10^−9^	↑
**12**	25.1		Unknown	341.0528		89.0242 59.0138 71.0137	1.98371	2.6 × 10^−8^	2.2 × 10^−8^	↑
**13**	29.0		Unknown	242.0787		78.9588 168.0424	0.82795	0.43160	1.35421	↓

^#^ Metabolite number. * Confirmed with standard ↑ The level of the compound increases with roasting. ↓ The level of the compound decreases with roasting.

**Table 3:** MS/MS fragmentation of potential markers of coffee roasting process in positive ionization mode.

							VIP Values			
#	RT (min)	Molecular Feature	Tentative Identification	[M + H]^+^	Mass Error (ppm)	Main MS/MS Fragments	GCB vs. LRC	GCB vs. MRC	GCB vs. DRC	Roasting Trend
**1**	12.3		Unknown	177.0561		117.0327 89.0377 145.0268	1.719010	2.00514	1.60664	↓
**2**	14.9		Unknown	679.5175		-	1.69434	1.90782	1.79200	↓
**3**	18.3		Unknown	393.0609		38.9629	1.70296	1.80418	0.60806	-
**4**	18.3		Unknown	163.0394		89.0380 117.0327 135.0431	1.71644	1.86415	0.51289	-
**5**	19.1	C_7_H_13_NO_2_	*N*-methylpipecolic acid	144.1012	6	58.0649 84.0797 98.0973	1.71129	1.56815	1.76873	↓
**6**	19.4	C_5_H_11_NO_2_	Betaine *	118.0858	10	58.0646	1.76821	2.079	1.8509	↓
**7**	19.9	C_7_H_13_NO_2_	Proline betaine *	144.1019	6	98.0958 58.0637 84.0814	1.56002	1.30522	1.66228	↓
**8**	21.6		Unknown	295.1666		121.0275 175.1433 84.0804	1.53231	1.95759	1.7594	-
**9**	22.4	C_5_H_9_NO_2_	Proline *	116.0703	2	70.0641	1.53958	3.55 × 10^−8^	1.49 × 10^−8^	↓
**10**	23.5		Unknown	200.1220		-	0.40495	1.77901	1.77808	↓
**11**	23.5		Unknown	244.1122		141.0470 126.0236	0.31289	1.88784	1.80463	↓
**12**	23.5	C_6_H_8_N_2_S	2-Methyl-(methylthiol)pyrazine isomer	141.0483	10	126.0239 99.0134 82.0522	1.29913	1.81396	1.83413	↓
**13**	23.8		Unknown	236.1503		58.0647 57.0329 59.0723	1.7175	2.04337	1.86042	↓
**14**	24.2		Unknown	520.1954		123.0905 296.1594	0.986242	0	5.79 × 10^−9^	-
**15**	24.3		Unknown	266.1605		104.1064 60.0800	1.4989	1.60253	1.29524	↓
**16**	24.7		Unknown	226.1186		123.0915 110.0830	1.64839	1.91133	1.67194	↑
**17**	25.6		Unknown	129.0654		-	1.76951	9.33 × 10^−9^	3.66 × 10^−8^	↓
**18**	27.8		Unknown	198.1236		-	1.7959	2.11365	1.87729	↓
**19**	28.8	C_5_H_14_NO	Choline *,^a^	104.1065	7	58.0647 60.0804 45.0328	1.63786	0.18465	1.78252	↓
**20**	28.8		Unknown	258.1106		104.1070 124.9995 86.0961 184.0730	1.53731	0.15982	1.77857	↓

^#^ Metabolite number. * Confirmed with standard. ^a^ [M]^+^, ↑ The level of the compound increases with roasting. ↓ The level of the compound decreases with roasting.

## Supplementary Materials

The following are available online, Figure S1: Base peak chromatograms (BPC) obtained in positive ionization mode for green coffee (GCB) (A); light coffee (LRC) (B); medium coffee (MRC) (C); and dark coffee (DRC) (D) under optimal separation conditions. HILIC-MS conditions are summarized in Section 3.3., Figure S2: Hierarchical cluster analysis (HCA) in positive (A) and negative (B) ionization modes for the four groups of coffee samples (GCB, LRC, MRC, and DRC) submitted to different roasting, Figure S3: Diagrams of the trends observed for all the tentatively and unequivocally compounds both in negative and positive ionization mode along the coffee roasting process.
