# Supplementary material for: Untargeted HILIC-MS-Based Metabolomics Approach to Evaluate Coffee Roasting Process: Contributing to an Integrated Metabolomics Multiplatform
Source: Molecules. 2020 Feb 17;25(4):887. doi: 10.3390/molecules25040887 (PMC7070313; doi:10.3390/molecules25040887)
Supplement: Supplementary file 1 [file molecules-25-00887-s001.pdf]

UNTARGETED HILIC-MS-BASED METABOLOMICS APPROACH TO EVALUATE COFFEE ROASTING PROCESS: CONTRIBUTING TO AN INTEGRATED METABOLOMICS MULTIPLATFORM

Raquel Pérez-Míguez[1#], Maria Castro-Puyana[1,2#], Elena Sánchez-López[1,2], Merichel Plaza[1,2], Maria Luisa Marina[1,2*].

[1]Departamento de Química Analítica, Química Física e Ingeniería Química, Universidad de Alcalá, Ctra. Madrid-Barcelona Km. 33.600, 28871 Alcalá de Henares (Madrid), Spain.

[2]Instituto de Investigación Química "Andrés M. del Río" (IQAR), Universidad de Alcalá, Ctra. Madrid-Barcelona Km. 33.600, 28871 Alcalá de Henares (Madrid), Spain.

[#]These authors contributed equally to this work.

***Correspondence:** mluisa.marina@uah.es; Fax: +34-918854971; Tel.: +34-918854935 (Departamento de Química Analítica, Química Física e Ingeniería Química, Universidad de Alcalá, Ctra. Madrid-Barcelona Km. 33.600, 28871 Alcalá de Henares (Madrid), Spain).

Figure captions.

Figure S1. Base peak chromatograms (BPC) obtained in positive ionization mode for green coffee (GCB) (A); light coffee (LRC) (B); medium coffee (MRC) (C); and dark coffee (DRC) (D) under optimal separation conditions. HILIC-MS conditions are summarized in section 3.3.

Figure S2. Hierarchical cluster analysis (HCA) in positive (A) and negative (B) ionization modes for the four groups of coffee samples (GCB, LRC, MRC, and DRC) submitted to different roasting process.

Figure S3. Diagrams of the trends observed for all the tentatively and unequivocally compounds both in negative and positive ionization mode along the coffee roasting process.

Figure S1.

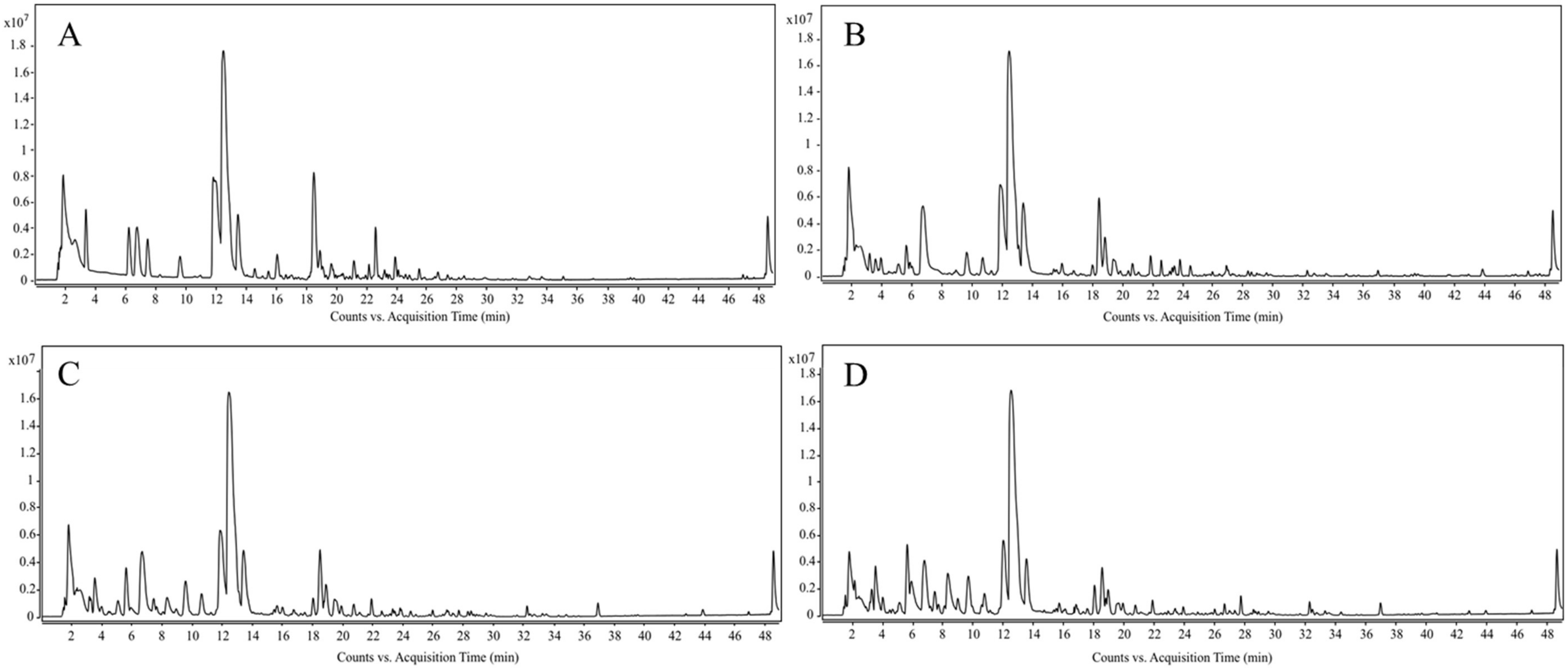
A
x10^7
1.8
1.6
1.4
1.2
1
0.8
0.6
0.4
0.2
0
2 4 6 8 10 12 14 16 18 20 22 24 26 28 30 32 34 36 38 40 42 44 46 48
Counts vs. Acquisition Time (min)
B
x107
1.8
1.6
1.4
1.2
1
0.8
0.6
0.4
0.2
0
2 4 6 8 10 12 14 16 18 20 22 24 26 28 30 32 34 36 38 40 42 44 46 48
Counts vs. Acquisition Time (min)
C
x107
1.6
1.4
1.2
1
0.8
0.6
0.4
0.2
0
2 4 6 8 10 12 14 16 18 20 22 24 26 28 30 32 34 36 38 40 42 44 46 48
Counts vs. Acquisition Time (min)
D
x107
1.8
1.6
1.4
1.2
1
0.8
0.6
0.4
0.2
0
2 4 6 8 10 12 14 16 18 20 22 24 26 28 30 32 34 36 38 40 42 44 46 48
Counts vs. Acquisition Time (min)

Figure S2-A

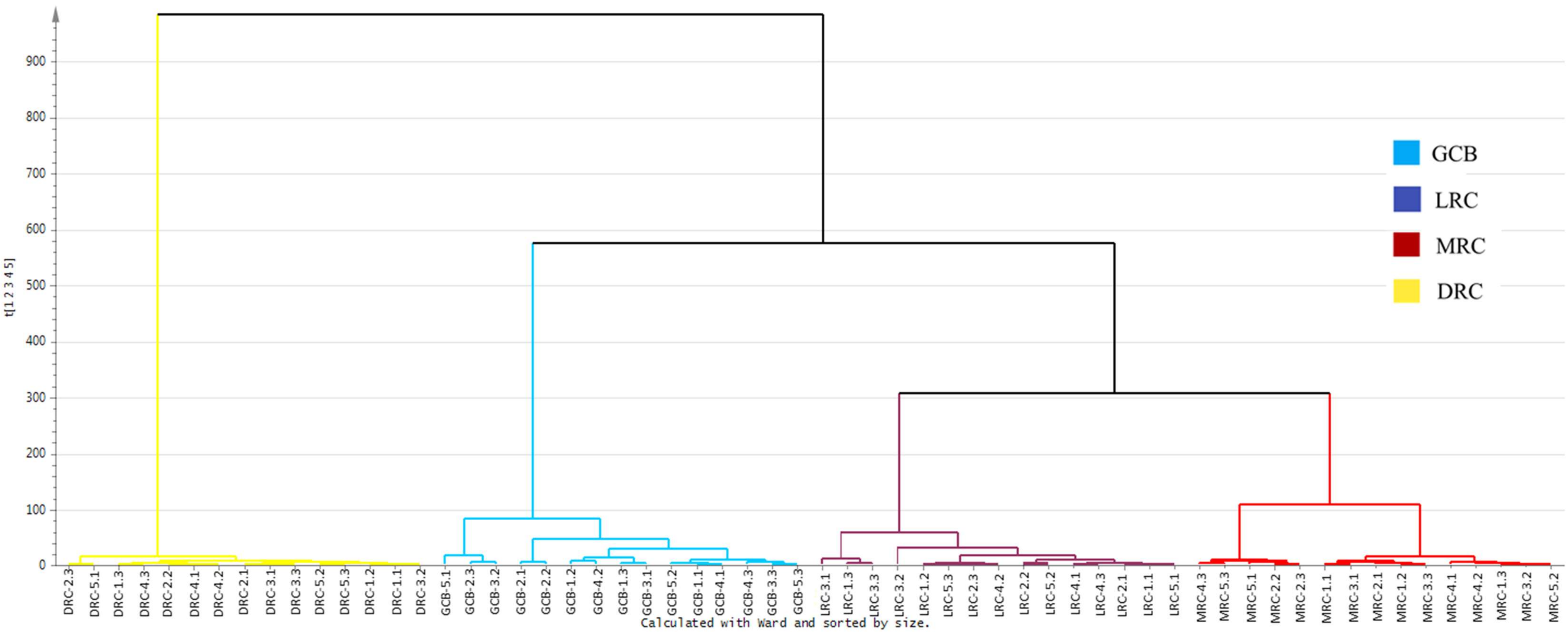
GCB
LRC
MRC
DRC
t[1 2 3 4 5]
0
100
200
300
400
500
600
700
800
900
DRC-2.3 DRC-5.1 DRC-1.3 DRC-4.3 DRC-2.2 DRC-4.1 DRC-4.2 DRC-2.1 DRC-3.1 DRC-3.3 DRC-5.2 DRC-5.3 DRC-1.2 DRC-1.1 DRC-3.2
GCB-5.1 GCB-2.3 GCB-3.2 GCB-2.1 GCB-2.2 GCB-1.2 GCB-4.2 GCB-1.3 GCB-3.1 GCB-5.2 GCB-1.1 GCB-4.1 GCB-4.3 GCB-3.3 GCB-5.3
LRC-3.1 LRC-1.3 LRC-3.3 LRC-3.2 LRC-1.2 LRC-5.3 LRC-2.3 LRC-4.2 LRC-2.2 LRC-5.2 LRC-4.1 LRC-4.3 LRC-2.1 LRC-1.1 LRC-5.1
MRC-4.3 MRC-5.3 MRC-5.1 MRC-2.2 MRC-2.3 MRC-1.1 MRC-3.1 MRC-2.1 MRC-1.2 MRC-3.3 MRC-4.1 MRC-4.2 MRC-1.3 MRC-3.2 MRC-5.2
Calculated with Ward and sorted by size.

Figure S2-B

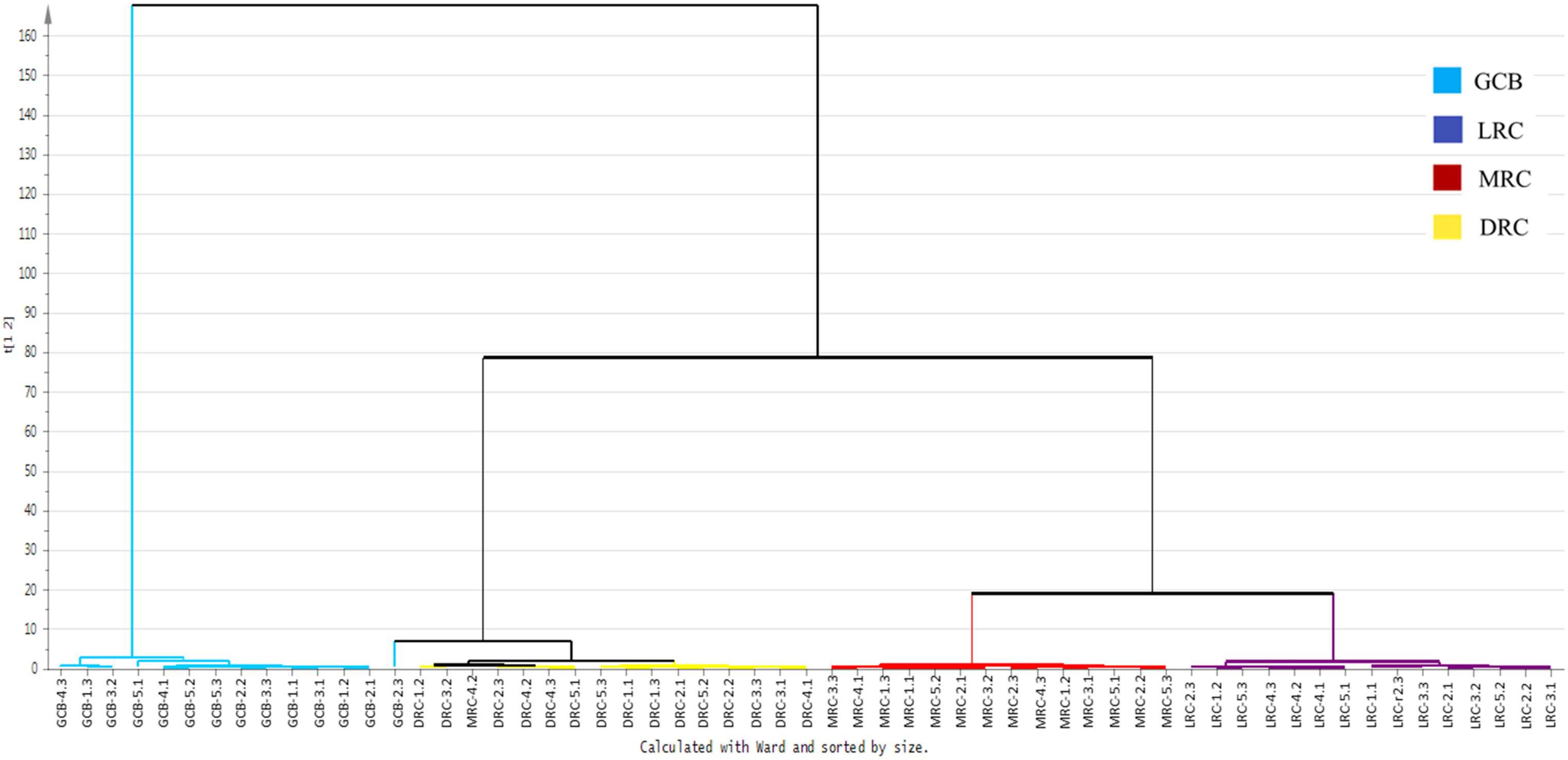
GCB
LRC
MRC
DRC
t[1 2]
160
150
140
130
120
110
100
90
80
70
60
50
40
30
20
10
0
GCB-4.3 GCB-1.3 GCB-3.2 GCB-5.1 GCB-4.1 GCB-5.2 GCB-5.3 GCB-2.2 GCB-3.3 GCB-1.1 GCB-3.1 GCB-1.2 GCB-2.1 GCB-2.3 DRC-1.2 DRC-3.2 MRC-4.2 DRC-2.3 DRC-4.2 DRC-4.3 DRC-5.1 DRC-5.3 DRC-1.1 DRC-1.3 DRC-2.1 DRC-5.2 DRC-2.2 DRC-3.3 DRC-3.1 DRC-4.1 MRC-3.3 MRC-4.1 MRC-1.3 MRC-1.1 MRC-5.2 MRC-2.1 MRC-3.2 MRC-2.3 MRC-4.3 MRC-1.2 MRC-3.1 MRC-5.1 MRC-2.2 MRC-5.3 LRC-2.3 LRC-1.2 LRC-5.3 LRC-4.3 LRC-4.2 LRC-4.1 LRC-5.1 LRC-1.1 LRC-r2.3 LRC-3.3 LRC-2.1 LRC-3.2 LRC-5.2 LRC-2.2 LRC-3.1
Calculated with Ward and sorted by size.

Figure S3.

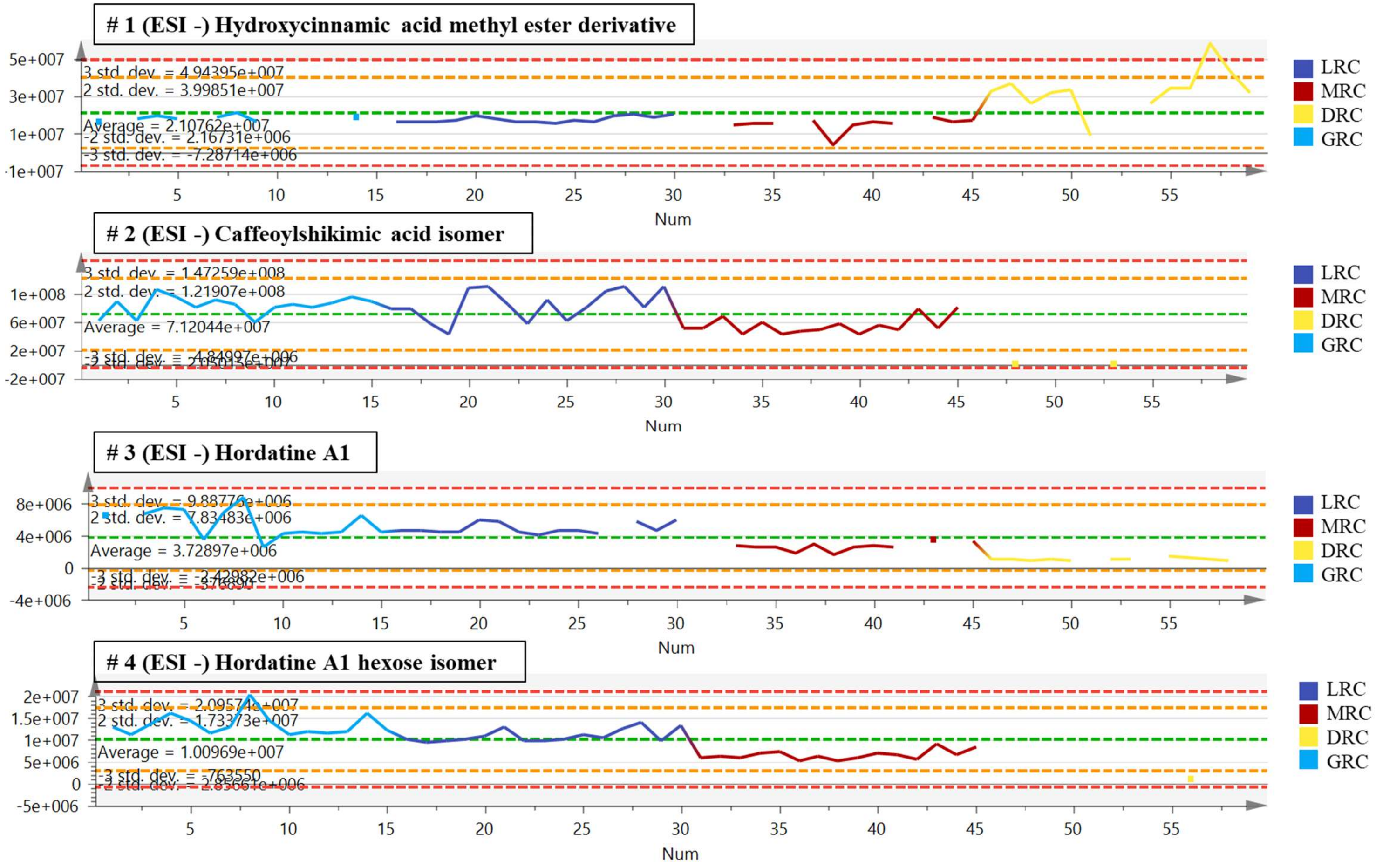

1 (ESI -) Hydroxycinnamic acid methyl ester derivative
3 std. dev. = 4.94395e+007
2 std. dev. = 3.99851e+007
Average = 2.10762e+007
-2 std. dev. = 2.16731e+006
-3 std. dev. = -7.28714e+006
Num
LRC
MRC
DRC
GRC
2 (ESI -) Caffeoylshikimic acid isomer
3 std. dev. = 1.47259e+008
2 std. dev. = 1.21907e+008
Average = 7.12044e+007
Num
LRC
MRC
DRC
GRC
3 (ESI -) Hordatine A1
2 std. dev. = 7.83483e+006
Average = 3.72897e+006
Num
LRC
MRC
DRC
GRC
4 (ESI -) Hordatine A1 hexose isomer
2 std. dev. = 1.73373e+007
Average = 1.00969e+007
Num
LRC
MRC
DRC
GRC

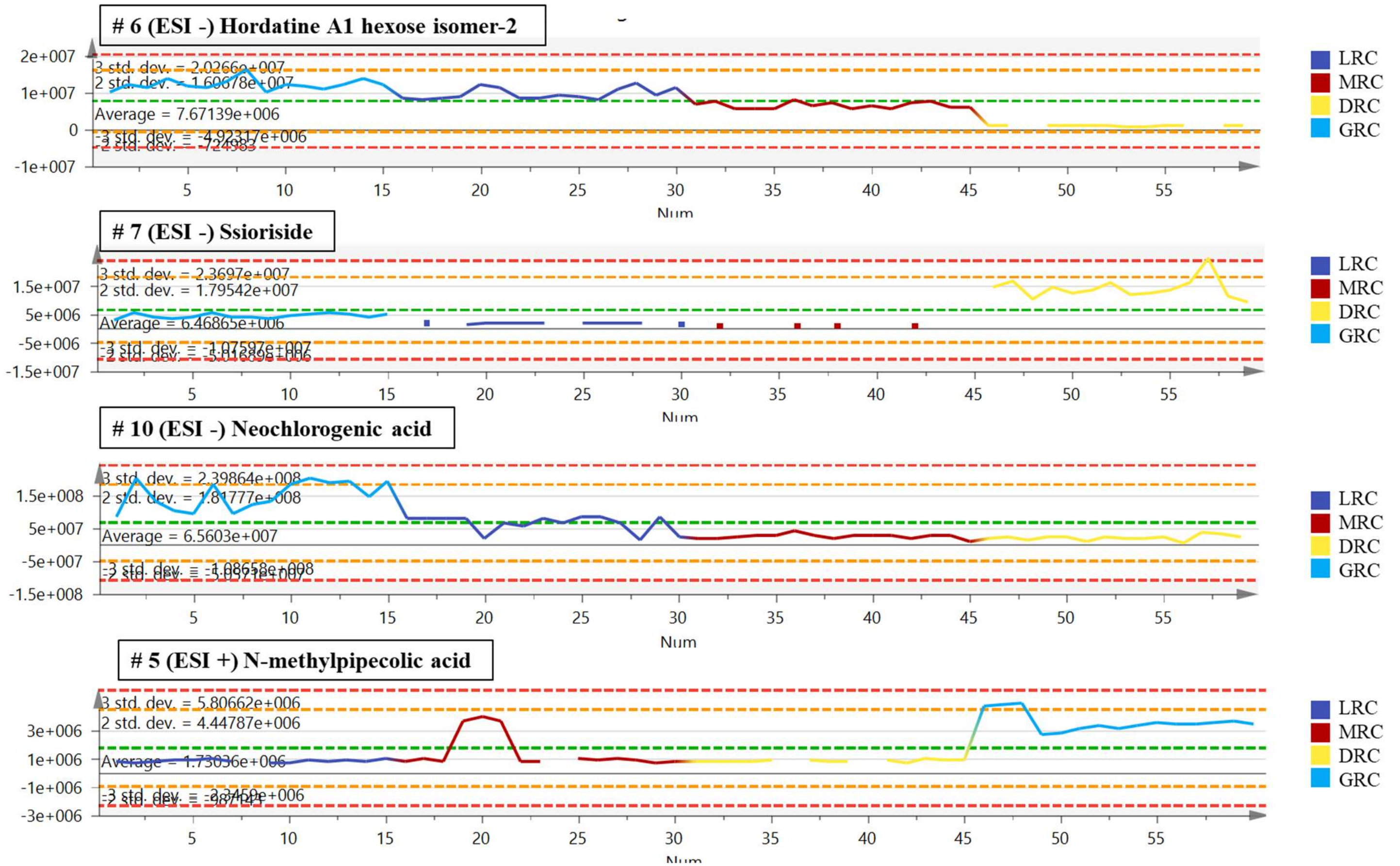

# 6 (ESI -) Hordatine A1 hexose isomer-2
Average = 7.67139e+006
Num
7 (ESI -) Ssioriside
3 std. dev. = 2.3697e+007
2 std. dev. = 1.79542e+007
Num
10 (ESI -) Neochlorogenic acid
3 std. dev. = 2.39864e+008
2 std. dev. = 1.81777e+008
Average = 6.5603e+007
Num
5 (ESI +) N-methylpipecolic acid
3 std. dev. = 5.80662e+006
2 std. dev. = 4.44787e+006
LRC
MRC
DRC
GRC

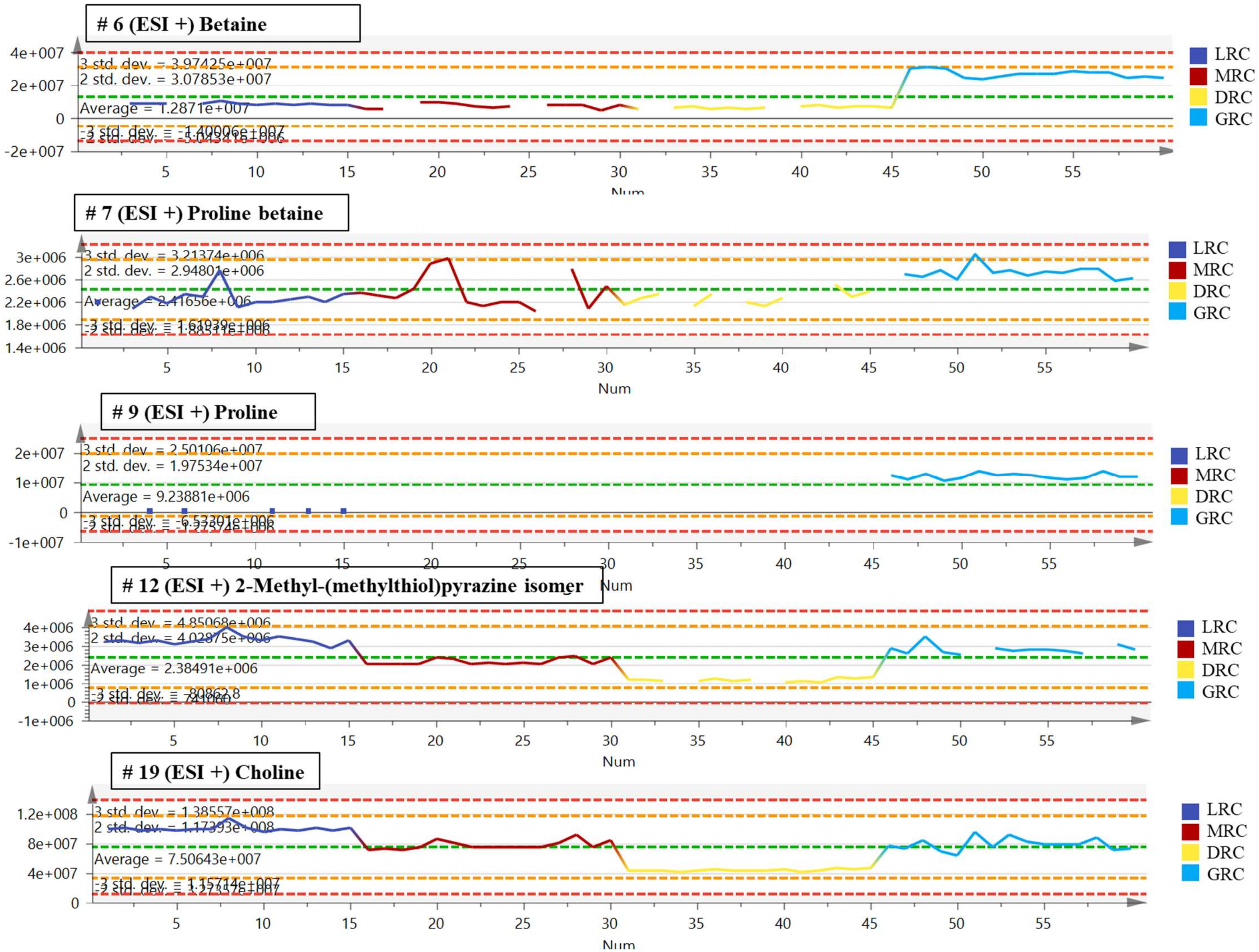

6 (ESI +) Betaine
3 std. dev. = 3.97425e+007
2 std. dev. = 3.07853e+007
Average = 1.2871e+007
4e+007
2e+007
0
-2e+007
Num
7 (ESI +) Proline betaine
3 std. dev. = 3.21374e+006
2 std. dev. = 2.94801e+006
Average = 2.41656e+006
3e+006
2.6e+006
2.2e+006
1.8e+006
1.4e+006
Num
9 (ESI +) Proline
3 std. dev. = 2.50106e+007
2 std. dev. = 1.97534e+007
Average = 9.23881e+006
2e+007
1e+007
0
-1e+007
Num
12 (ESI +) 2-Methyl-(methylthiol)pyrazine isomer
3 std. dev. = 4.85068e+006
2 std. dev. = 4.02875e+006
Average = 2.38491e+006
4e+006
3e+006
2e+006
1e+006
0
-1e+006
Num
19 (ESI +) Choline
3 std. dev. = 1.38557e+008
2 std. dev. = 1.17393e+008
Average = 7.50643e+007
1.2e+008
8e+007
4e+007
0
Num
LRC
MRC
DRC
GRC
